# **Full-color micro**-LED** display with photo-patternable and highly ambient-stable perovskite quantum dot/siloxane composite as color conversion layers**

**DOI:** 10.1038/s41598-023-31945-6

**Published:** 2023-03-24

**Authors:** Hyung Cheoul Shim, Juho Kim, So Yeon Park, Bong Sung Kim, Bongkyun Jang, Hak-Joo Lee, Areum Kim, Seungmin Hyun, Jae-Hyun Kim

**Affiliations:** 1 Department of Nano-Mechanics, Korea Institute of Machinery & Materials (KIMM), 156, Gajeongbuk-ro, Yuseong-gu, Daejeon, 34103 Republic of Korea; 2 Department of Nanomechatronics, University of Science and Technology (UST), Daejeon, 34113 Republic of Korea; 3 Center for Advanced Meta-Materials (CAMM), 156 Gajeongbuk-Ro, Yuseong-gu, Daejeon, 34103 Republic of Korea

**Keywords:** Quantum dots, Inorganic LEDs

## Abstract

In this paper, we successfully fabricated color conversion layers (CCLs) for full-color-mico-LED display using a perovskite quantum dot (PQD)/siloxane composite by ligand exchanged PQD with silane composite followed by surface activation by an addition of halide-anion containing salt. Due to this surface activation, it was possible to construct the PQD surface with a silane ligand using a non-polar organic solvent that does not damage the PQD. As a result, the ligand-exchanged PQD with a silane compound exhibited high dispersibility in the siloxane matrix and excellent atmospheric stability due to sol–gel condensation. Based on highly ambient stable PQD/siloxane composite based CCLs, full-color micro-LED display has a 1 mm pixel pitch, about 25.4 pixels per inch (PPI) resolution was achieved. In addition, due to the thin thickness of the black matrix to prevent blue light interference, the possibility of a flexible display that can be operated without damage even with a bending radius of 5 mm was demonstrated.

## Introduction

Quantum dots (QDs) is one of the materials attracting attention as a candidate for color conversion layers (CCLs) of mirco-LED because of its excellent photophysical characteristics that can provide high color gamut compared to conventional fluorescent phosphors^1,2^. However, due to the surface defects of QDs originated from their intrinsic large surface-to-volume ratio, thick shelling is needed to preserve the unique optical properties of QDs, which requires an additional complicated synthesis process. Perovskite quantum dots (PQDs), which exhibits defect tolerant properties compared to conventional QDs^3^, may have an advantage over QD in this respect. In other words, PQDs can provide high photoluminescence quantum yield (PLQY) and narrow emission without shelling, allowing for facile and simple synthesis.

Therefore, the PQDs can be considered more promising as a display material that can express stable and high luminescence performance with low cost as compared to conventional covalent bond based QDs^4–9^. For this reason, PQD has recently been attracting attention as a material for CCLs^10–12^. PQD CCLs have been implemented using a variety of techniques, including the most prominent options such as spin coating^13^, ink-jet printing^14,15^ and vacuum drying^16^. In order to achieve efficient CCLs, the energy of the backlight unit with short wave length located at the bottom of the CCLs, should be absorbed as much as possible for photoconversion without loss. Therefore, a structure that is sufficiently thick in the micrometer unit needs to be used, however, which is often challenging to do using spin coating^12^.

In the case of ink-jet printing or vacuum drying, interlayer defects or surface uniformity problems may occur during the repeated process to build up a thick layer. In addition, since PQD is known to be very sensitive to oxygen or moisture in the air^17^, it is required to improve the atmospheric stability through the composite with a polymer^18,19^ or encapsulation^20^. However, the dispersibility issue of the polymer matrix may have an impact on the photoluminescence (PL) efficiency of PQDs when they are combined with polymers^21,22^. Moreover, polar organic solvents used to mainly disperse the polymer can potentially cause decomposition of PQDs^23,24^.

In this paper, we used the squeezing technique to fabricate PQD CCLs for Miro-LED that have sufficient thickness and can be easily photopatterned. Above all, excellent dispersibility in the siloxane matrix was induced without damage to the PQD by applying a method of activating the PQD surface using anion salt and then arranging a silane ligand. In addition, based on the sol-gel condensation reaction, the PQD was well encapsulated in the siloxane matrix, and it was possible to fabricate full color CCLs for micro-LED with excellent atmospheric stability that maintains 80 % of the original PL efficiency even after 1 month. Since this technique is a process that can be applied to very flexible PCB substrates, we were able to demonstrate a flexible full-color micro-LED that can operate without any problem even with a bending radius of 5 mm.

## Experimental details

### Synthesis of oleate-perovskite quantum dots (oleate-PQDs, CsPbBr_3_, CsPbI_3_)

For the preparation of Cs-oleate solution, Cs_2_CO_3_ (0.407 g), oleic acid (OA, 1.25 mL), and 1-octadecene (ODE, 25 mL) were added to a three-necked flask and then stirred in a vacuum atmosphere while heated to 120 °C for 30 minutes. After the complete dissolution of metal salt, the solution was purged with nitrogen (N_2_), and its temperature was raised up to 135 °C. To prepare an oleate mixture for a Pb-oleate solution, OA and oleylamine (OAm) were mixed in a 1:1 (*v*/*v*) ratio and placed in a three-necked flask followed by stirring in a vacuum atmosphere, heating at 120 °C for 30 minutes. After the oleate mixture was purged with N_2_, and the flask was heated up to 130 °C. For the preparation of Pb-oleate solution, 1.2 mmol of PbBr_2_ and ZnBr_2_ powder (PbI_2_ and ZnI_2_ for the CsPbI_3_) was dissolved in 25 mL of ODE in a flask powder (50 mL of ODE for the CsPbI_3_). This mixture was also subjected to a vacuum for 30 minutes and heated up to 120 °C. After N_2_ purging, preheated 7.5 mL of the oleate solution (a mixture of OA and OAm) was added to the Pb-oleate solution. After the reaction was complete and the solution became transparent, the mixed solution was heated up to 170 °C. Then, 3 mL of the Cs-oleate solution was rapidly injected and maintained for 10 seconds, and then instantly cooled down to room temperature using ice water to synthesize perovskite quantum dots (CsPbBr_3_, CsPbI_3_) with oleate ligand (oleate-PQDs). In order to purify the synthesized oleate-PQDs crude solution, the colloidal solution was centrifuged with adding methyl acetate (MeOAc) and redispersed in hexane. After removing excessive oleate and metal salts through repeated centrifugation and redispersion, the PQD solution was stabilized in a refrigerator for at least 24 hours.

### Preparation of the Perovskite QDs/Siloxane resin

#### Ligand exchange process for silane-PQDs

After mixing of oleate-PQDs solutions (10 mg/mL) dispersed in toluene (10 mL), methylammonium bromide (0.1 g), and 20 µL of (3-mercaptopropyl)methyl-dimethoxy silane, the mixture was stirred in an N_2_ purged atmosphere for 1 hour for the ligand exchange with silane. Unreacted silane and halogen compounds were removed by centrifugation at 12,000 rpm for 30 minutes, and the precipitated nanoparticles were dispersed in toluene.

#### Preparation of silane-PQDs/siloxane resin

3-(trimethoxysilyl)propyl methacrylate (MPTMS) and diphenylsilanediol (DPSD) were added to a 250 mL 2-neck flask at a molar ratio of 1:1, and then barium hydroxide monohydrate (Ba(OH)_2_∙H_2_O) as a catalyst was added with an amount of 0.1 mol % of MPTMS. The mixture was stirred at 85 °C for 5 hours to induce a sol-gel condensation reaction to obtain methacrylate oligosiloxane resins. After completing the reaction, the clear resin was stirred at room temperature under a vacuum to remove methanol that may be produced as a byproduct. Then, after cooling to room temperature with N_2_ purging, 50 mL of the silane-PQD solution was mixed with siloxane resins (siloxane resin: PQD = 100:1, wt %) and stirred for 2 hours until all of the organic solvents were volatilized. Next, a photocuring catalyst (2,2-dimethoxy-2-phenyl acetophenone) was added to the silane-PQD/siloxane resin mixture in an amount of 0.2 wt % of the total siloxane composite mass. For the characterization of silane-PQD/siloxane resin, the composite was put in a mold and then exposed to ultraviolet light with a wavelength of 365 nm for 10 minutes to obtain a cured resin specimen.

### Characterization of the Perovskite QDs/Siloxane resin

Transmission electron microscopy (TEM) images of PQDs were obtained using a Tecnai F30 Super-Twin (FEI). The absorption and photoluminescence (PL) spectra of PQD samples were measured using a UV-Vis spectrometer (Shimadzu, UV3600) and a PL spectrometer (Fluorolog, Horiba Jobin Yvon Inc.). Fourier transform infrared (FTIR) spectroscopy analysis was performed using a Thermo Nicolet 6700.

### Preparation of the blue cutting film

The fabrication procedures and optical microscope images are illustrated in Fig. S3. First, a metal mask (Ni, 200 nm) was deposited on a PI (Polyimide) film (~50 μm) by an e-beam evaporator and a lift-off process. A dry etching (75W, 150 mTorr, 10 sccm O_2_) through the metal mask produced hole arrays for blue pixels. After removing the metal mask using an etchant (1:1:5 mixture of HCl, H_2_O_2_, and H_2_O), sputtering and lifting off a metal film (Cr, 150 nm) on the PI film form a black matrix layer.

### Transfer of micro-LED array onto a flexible film

A SU-8 (Kayaku Advanced Materials Inc.) mold for a patterned PDMS (Polydimethylsiloxane) stamp (60 µm × 100 µm, thickness: ~70 µm) was fabricated on a silicon wafer using photolithography. After pouring a mixture of PDMS base and curing agent (sylgard184, Dow corning) on the SU-8 mold, the PDMS was cured at room temperature for 48 hours to prevent thermal deformation. The replicated PDMS stamp selectively picked up a micro-LED array from a donor substrate and placed the array onto a flexible PCB (Printed circuit board) film (polyimide, ~12.5 µm). Heating with a hot plate (243 °C) electrically interconnected micro-LEDs and electrodes on the flexible PCB film through solder.

### Fabrication of the full-color PQD micro-LED display

A black photoresist (~20 µm, GMC1070, Gersteltec) was spin-coated and degassed to fill the air gap between the micro-LED and the flexible film, followed by photolithographic openings to the micro-LEDs, as shown in Fig. S2a. Another patterning of the black photoresist (~40 µm, GMC1070, Gersteltec) forms partition walls to block light leakage (Fig. S2c). The green PQD resin was dispensed onto the micro-LED array, followed by degassing to fill the partition walls with the resin. Then, the surplus resin was removed by a squeegee. UV exposure through a mask selectively cured the PQD resin. The uncured PQD resin was cleaned with acetone. The PL spectra revealed that the acetone cleaning procedure had no impact on the optical characteristics of PQD resin film (Fig. S4). The squeegee photopatterning was once again performed using the red PQD resin to prepare red subpixels. An adhesive layer (sylgard184, Dow corning) was spin-coated and cured at 70 °C for 2 hours.

### Optical characterization of the full-color PQD micro-LED display

The fluorescence spectra of the PQD films with various thicknesses (~24 µm, ~44 µm, ~58 µm, ~73 µm) were measured using a UV–Vis spectrometer (Shimadzu, UV3600) with an excitation source (~365 nm). The spectra and CIE chromaticity coordinates of the PQD micro-LED display were measured using a spectroradiometer (CS-2000, Konica Minolta). PLQY was measured using absolute PL quantum yield measurement system (C9920-02, Hamamatsu, Japan) with a Xenon light source (150 W).

## Results and discussion

**Figure 1:**
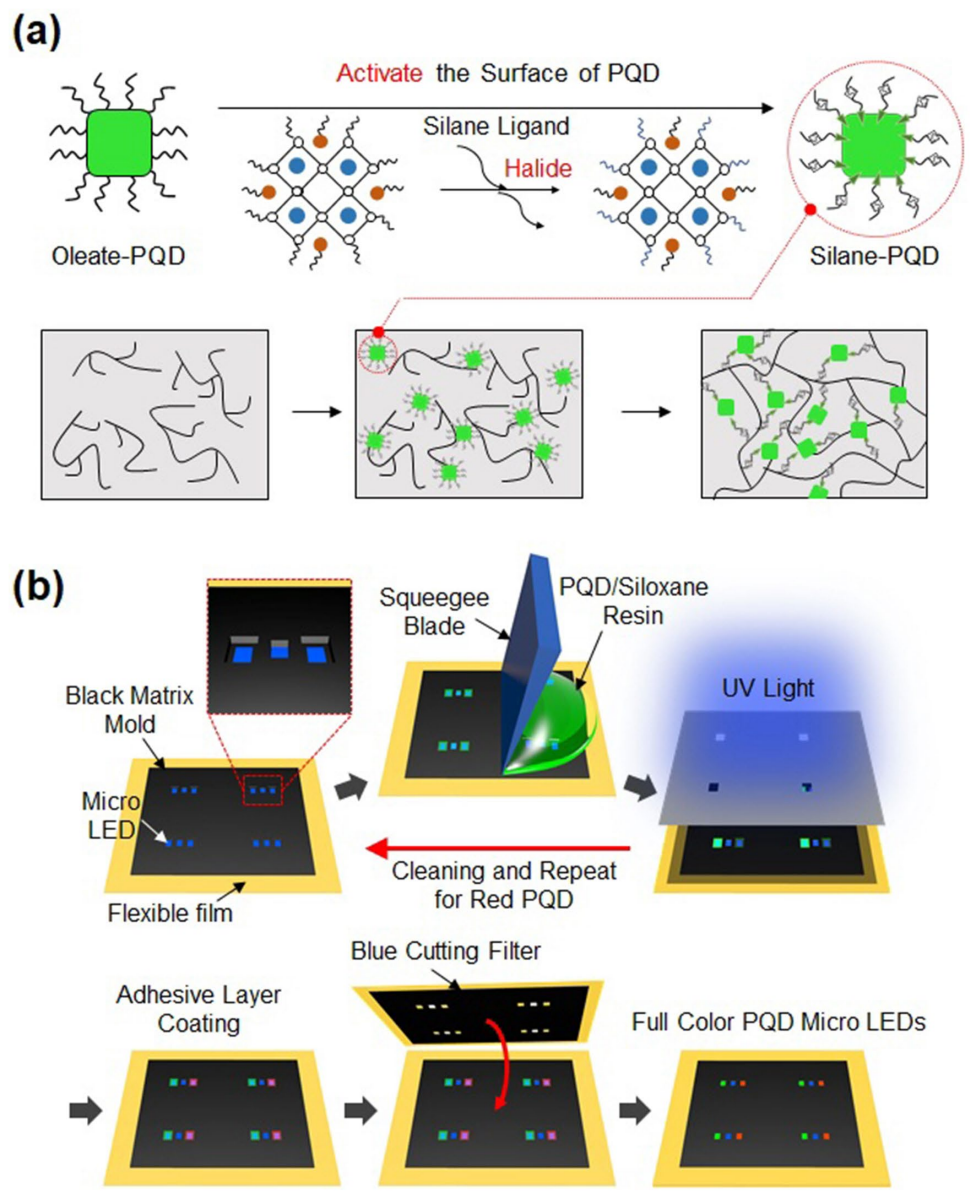
Schematic illustration of (**a**) ligand exchange of oleate-capped perovskite quantum dots (PQDs) with silane-capped PQDs and (**b**) fabrication procedure for full-color micro-LEDs with PQD/siloxane composite as color conversion layers.

Sol–gel-derived siloxane-based hybrid materials are promising candidates for the effective encapsulation of PQDs because they are highly transparent and have higher thermal stability than other polymers^25,26^. However, the hydrophobic nature of PQDs hinders their dispersion in the siloxane matrix^27^. To avoid using a general polar solvent that could decompose the PQDs, the dispersibility of PQDs in oligosiloxane-based hybrid materials was improved by replacing the PQD capping ligands, i.e. oleic acid (OA) and oleic amine (OAm), with silane ligands^28^. First, the PQD surface was activated by anion substitution, and the OA/OAm ligands surrounding the PQDs were then replaced with silane ligands. Specifically, as shown in Fig. 1a, the OA/OAm ligands could be replaced with a silane ligand without damaging the PQDs by activating the PQD surface using methylammonium bromide followed by mixing with 3-mercaptopropyl(methyl)dimethoxysilane. Since the OA/OAm ligands are loosely bound to the PQD surface^29^, this method benefits from the soft lattice properties of PQDs to successfully replace the original ligands of PQDs without changing the PQD structure using small amounts of silane compounds (less than 20 µL). Then, the silane ligands on the PQD surface formed a network structure via condensation of the silane groups in the siloxane resin.

A squeegee photopatterning method was employed to obtain a patterned PQD array with thermal stability and uniform dispersion during the patterning process for a full-color PQD micro-LED display. Figure 1b shows a schematic illustration of the method. First, micro-LEDs (100 µm × 60 µm, thickness ~ 7 µm) were prepared on a flexible PCB film (polyimide,  ~ 12.5 µm) using a transfer-printing technology and an adhesive layer. Subsequently, a mold structure was fabricated by regulating the thickness and lateral dimension of the PQD resin. The mold was patterned using a black photoresist (GMC1070, Gersteltec), forming a black matrix that constitutes an essential layer to prevent optical crosstalk in the display technology and attenuate the optical intensity of the light passing through it^30^. This attenuation lowers the optical crosstalk among neighboring micro-LEDs, providing a better color gamut in the display.

After forming the mold, the green subpixel was introduced by pouring and spreading green PQD resin onto the entire region of the micro-LEDs. Degassing was performed to remove bubbles and to fill the well of the mold with the PQD resin. The surplus resin above the mold was removed using a polyurethane squeegee, as illustrated in Fig. 1b. After the squeegee process, the PQD resin was only contained in the well of the mold. The PQD resin in the green subpixel position was selectively cured by irradiating with ultraviolet (UV) light through a mask, and the uncured PQD resin was cleaned with acetone. In this squeegee photopatterning method, the use of the black matrix layer as a mold allows avoiding thermal and spin-coating processes, and only simple equipment such as a squeegee and a conventional lithography exposure tool is required. For the introduction of the red subpixel, the squeegee photopatterning process was repeated using red PQD resin.

**Figure 2:**
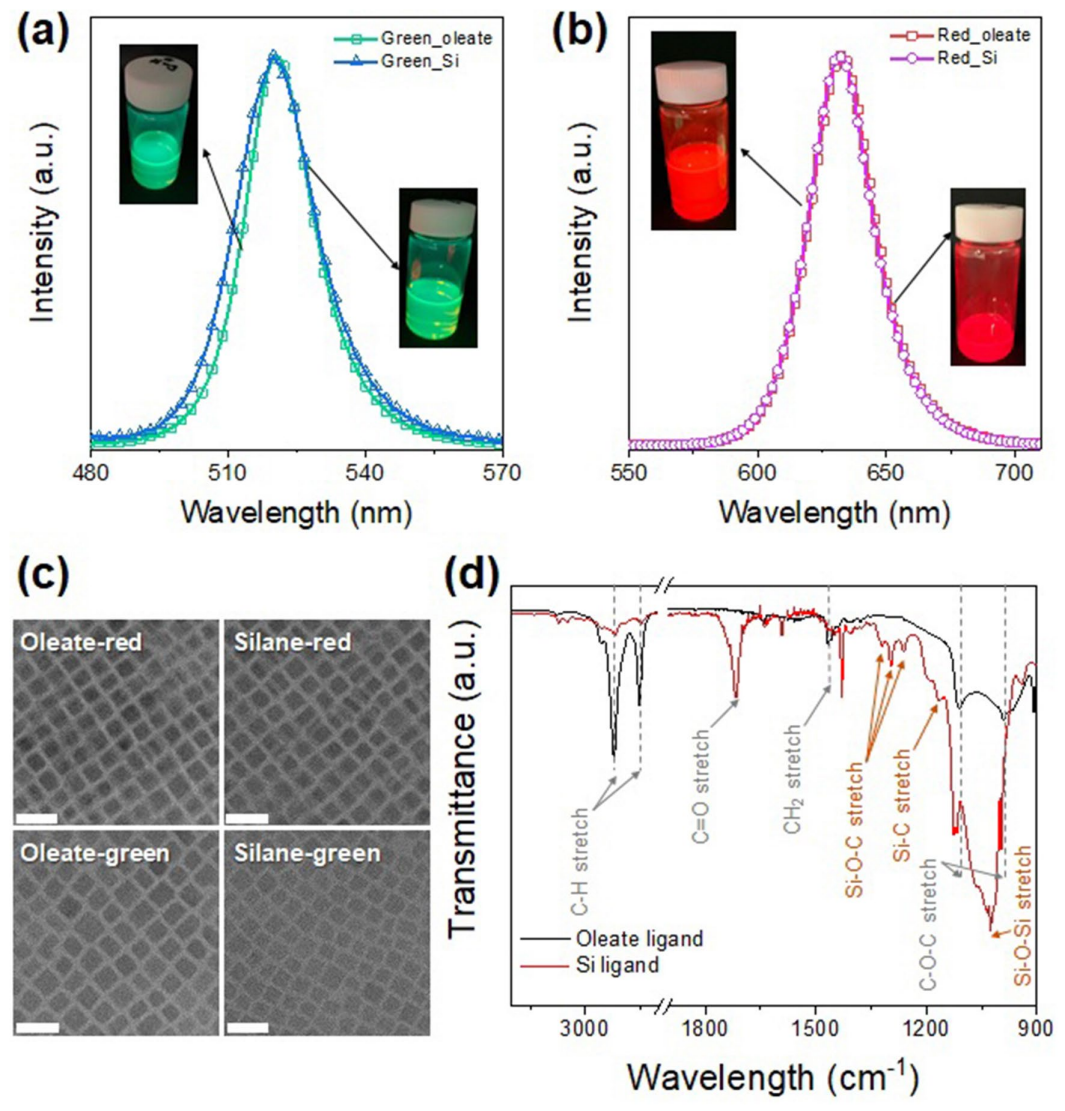
Photoluminescence (PL) spectra and transmission electron microscopy (TEM) images showing perovskite quantum dots (PQDs) with nearly identical morphologies and optical properties before and after ligand exchange with silane. PL spectra of (**a**) green PQDs with oleic acid (green solid line with hollow squares) and silane (blue solid line with hollow triangles) and (**b**) red PQDs with oleic acid (red solid line with hollow squares) and silane (pink solid line with hollow circles). (**c**) TEM images of green and red PQDs with oleic acid and silane (scale bar: 20 nm). (**d**) Fourier transform infrared (FTIR) spectra of oleate-capped PQDs (dark gray solid line) and silane-capped PQDs (red solid line). After the ligand exchange process, FTIR peaks attributable to silane compounds were detected.

The PL spectra shown in Fig. 2a and b reveal that the optical properties of the PQDs modified with silane ligands were virtually the same as those of the OA-capped PQDs. Of course, during ligand exchange, the PLQY of PQD solution slightly decreased from 88.5 to 84.1%, but it was still a high value (Table S1). Similarly, no change in the PQD morphology due to the ligand exchange process can be observed in the transmission electron microscopy images shown in Fig. 2c. Accordingly, it can be concluded that neither the ligand exchange process nor the silane ligand altered the optical and morphological properties of pristine PQDs.

**Figure 3:**
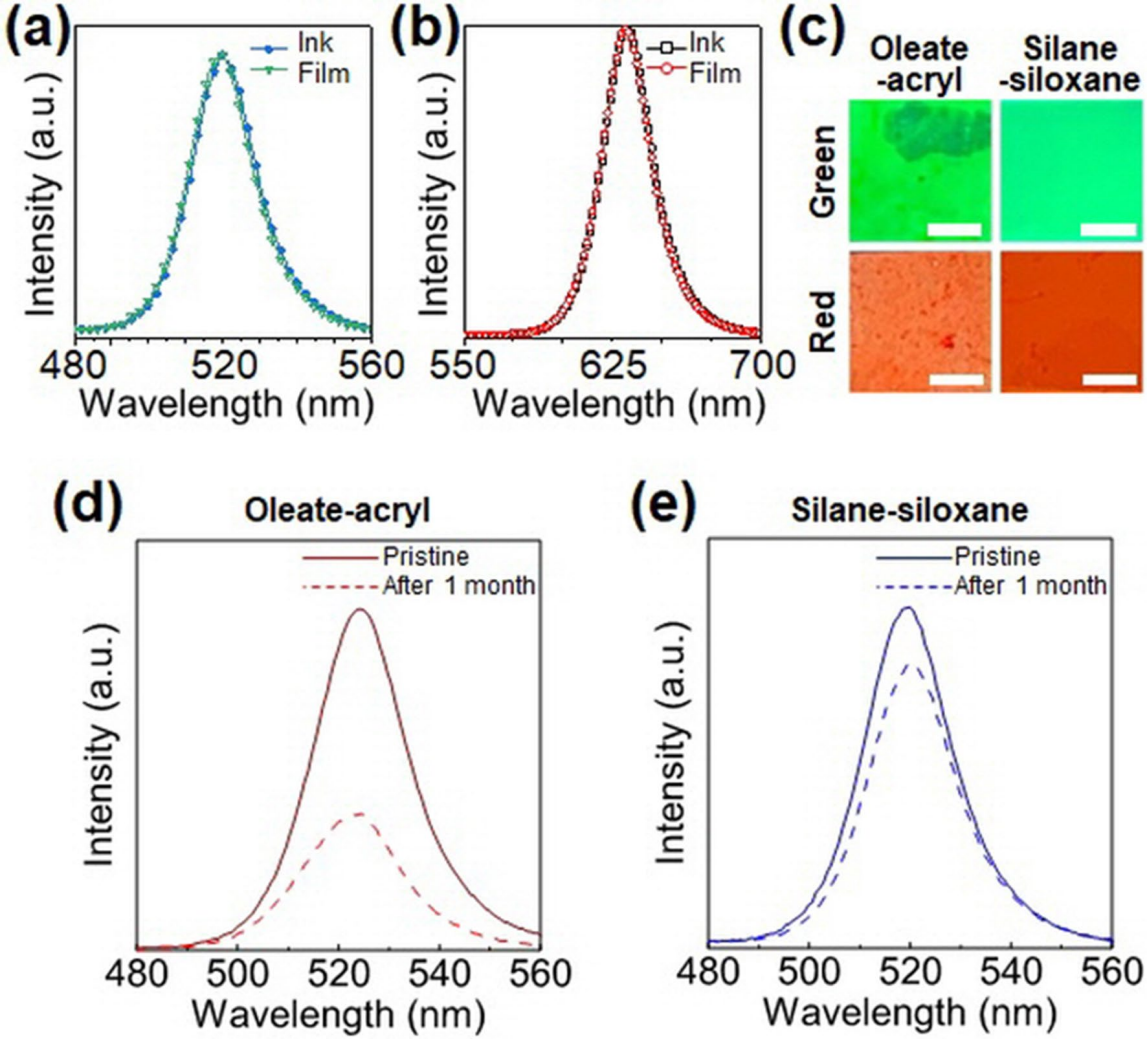
Photoluminescence (PL) spectra of (**a**) green perovskite quantum dots (PQDs) and (**b**) red PQDs in solution with silane and in film form after UV curing. (**c**) Optical microscope images of oleate-capped PQD/acrylate and silane-capped PQD/siloxane composites (scale bar: 500 μm). PL spectra of (**d**) oleate-capped PQD/acrylate composite and (**e**) silane-capped PQD/siloxane composite. The dash line indicates the PL spectra after one month of storage under ambient condition.

In addition, the Fourier transform infrared spectra shown in Fig. 2d confirm that the silane ligand successfully replaced the OA ligand on the pristine PQD surface. The PL spectra displayed in Fig. 3a and b show that the full width at half maximum (FWHM) and emission peak position of the PQD ink was unchanged after the film curing process using UV radiation. Taken together, these characterization results demonstrate that it was possible to fabricate a stable PQD/siloxane resin composite while preserving the optical characteristics of pristine PQDs.

The optical microscopic image shown in Fig. 3c reveals that there was no aggregation between PQDs in the PQD/siloxane resin composite. However, a significant aggregation between OA-capped PQDs was observed in the acrylate resin. In other words, when PQDs capped with OA ligand were dispersed in acrylic resin, the dispersibility of PQDs in the polymer matrix was lower than that of the PQD/siloxane composite containing PQDs with silane ligands. This is because the wettability between the oleate-rich PQD surface and the acrylate oligomer is relatively lower than that between the silane-capped PQDs and the oligosiloxane matrix.

The long-term atmospheric stability of the PQD/siloxane composite was evaluated by storing it under air conditions for one month. As shown in Fig. 3d, the PL intensity of the OA-capped PQD/acrylic composite was significantly degraded after 1 month, whereas the silane-capped PQD/siloxane resin composite maintained about 83% of the initial PL intensity (Fig. 3e) (in case of red PQD/siloxane resin, the PL intensity retention is about 80% as shown in Fig. S5). This result demonstrates that the degradation of the PQD properties due to exposure to atmospheric conditions can be prevented by the encapsulation with silane ligand in the siloxane matrix. In addition, through an additional thermal stability test, we found that the PQD/siloxane composite has superior thermal stability compared to the OA-capped PQD/acrylic composite. That is, as can be seen in Fig. S7, the acrylic based composite soon dropped to 25% of the initial PLQY value whereas PQD/siloxane composite retained 83% of the PLQY value. The atmospheric and thermal stability of these silane-capped PQD/siloxane composites could stem from the uniform passivation of PQDs by the siloxane matrix, which could act as an amorphous SiO_2_-like passivation layer around the PQDs^31,32^.

**Figure 4:**
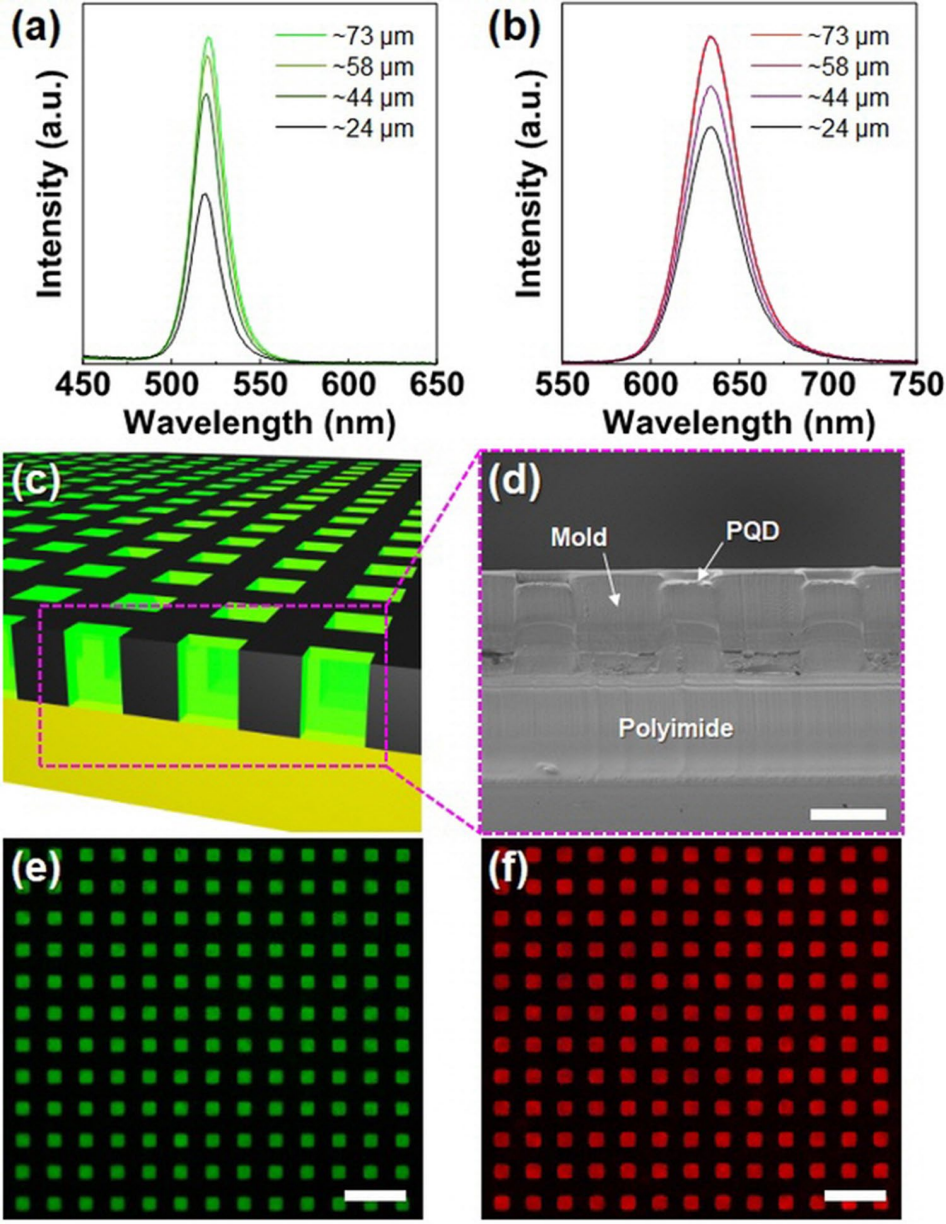
Photoluminescence spectra (PL) of (**a**) green and (**b**) red perovskite quantum dots (PQDs) with different thicknesses excited by blue LEDs (λ_ex_ = 460 nm). (**c**) Schematic illustration and (**d**) cross-sectional scanning electron microscopy image of photopatterned PQD/siloxane composite array filled with a black mold matrix (scale bar: 30 µm). Optical microscope images of photopatterned (**e**) green and (**f**) red PQD/siloxane composite array in a black mold matrix on a substrate, excited by blue LEDs (λ_ex_ = 460 nm) (scale bar: 100 µm).

Controlling the PQD thickness is essential for the light conversion efficiency and the absorbance of blue light as an excitation source for PQDs. Figure 4a and b present PL spectra of green and red PQD films with different film thicknesses (~ 24, ~ 44, ~ 58, and ~ 73 µm). As the thickness increases, it can be observed that the intensity of the converted light increases until saturation. These results agree with previous reports and indicate that an optimum thickness of the PQD layer is required for high conversion efficiency. However, the PLQYs of composite film decrease considerably as thickness increases (Fig. S8a). This phenomenon may be attributed to the aggregation of PQDs with increasing thickness or the loss of PL due to higher reabsorption as the film thickness increases related to the large spectral overlap of the absorption and emission spectra (Fig. S8b).

The advantage of the squeegee photopatterning process is that it allows controlling the width and thickness of the PQD pattern. Significantly, the thickness of the PQD pattern is directly related to the color conversion performance of the PQDs and can be controlled by changing the mold structures. Figure 4c shows a schematic illustration of a rectangular PQD pattern with a size of 30 µm designed to demonstrate the easy downscaling of the squeegee photopatterning method. The patterned PQD array has similar dimensions to the black matrix, as shown in the scanning electron microscopy image in Fig. 4d, which is another advantage of the proposed black matrix mold for the PQD pattern.

Although the resolution of our equipment limited the size of the rectangular patterns to 30 µm, smaller features could be achieved by increasing the resolution of the photopatternable black matrix. The PL images of the green and red PQD rectangular array excited by blue LEDs (~ 460 nm) are shown in Fig. 4e and f, respectively.

In our design, the PQDs convert the blue light emitted from blue micro-LEDs to red and green light, enabling RGB full-color display without additional transfer-printing process for red and green micro-LEDs. However, not all the blue light is converted to green or red light using the present PQDs. Fig. S1a and b shows the PL emission spectra of green and red subpixels without a blue filter, which reveal that a significant amount of blue light is transmitted through the PQDs without being converted. Several methods can be adopted to reduce the blue light passing through the PQDs, e.g., maximizing the PQD efficiency or increasing the PQD thickness and concentration. In this study, a polyimide (PI) film (50 µm) was used as a blue-light-cutting filter at the surface of the full-color micro-LED display. Since the PI film has a low transmittance below 500 nm, it can effectively reduce the intensity of transmitted blue light without requiring an additional filter such as a distributed Bragg reflector. Thus, the fabrication of the full-color PQD micro-LED display was completed by attaching a blue-light-cutting Cr/PI film (150 nm/50 µm) using a motorized microstage as illustrated in Fig. 1b. Details of the blue-light-cutting film process are described in the supplementary material.

**Figure 5:**
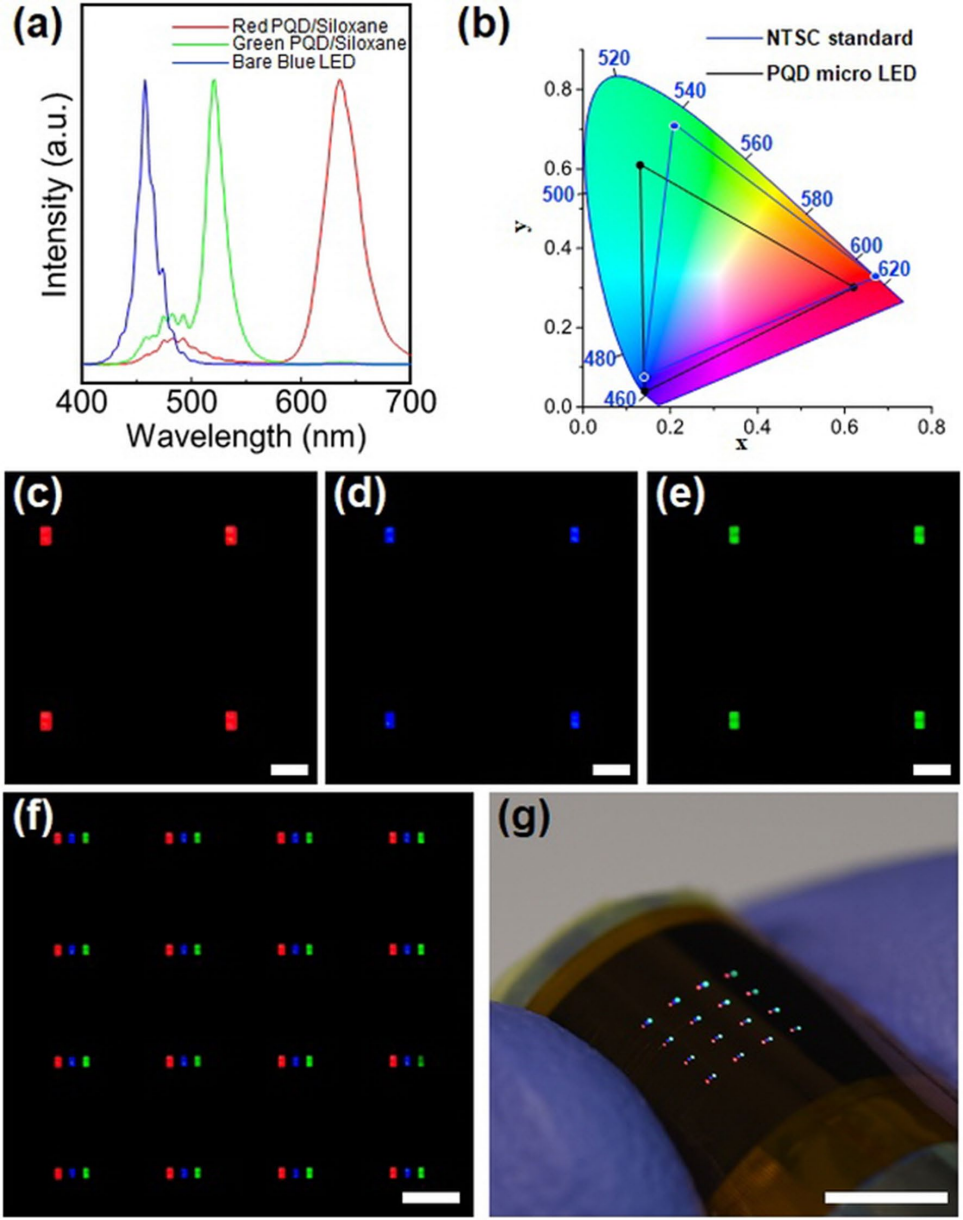
(**a**) Normalized photoluminescence spectra of RGB light from full-color micro-LEDs with perovskite quantum dot (PQD)/siloxane composite as color conversion layers (CCLs). (**b**) CIE color coordinates corresponding to the RGB light. The black and blue lines show the CIE color coordinates of the PQD micro-LED display and NTSC, respectively. Optical microscope images of (**c**–**e**) a partially enlarged individual pixel (scale bar: 200 µm) and (**f**) the whole pixels (scale bar: 500 µm) of the micro-LED with PQD/siloxane composite as CCLs. (**g**) Image of the full-color micro-LED with PQD/siloxane composite as CCLs under bending (scale bar: 5 mm).

Figure 5a presents the normalized PL spectra of RGB subpixels in the full-color micro-LED display, which shows that a considerable amount of blue light was reduced by the blue-light-cutting film. The CIE 1931 chromaticity coordinates of the PQD micro-LED display were measured and compared with the NTSC standard, finding that the color gamut of the micro-LED display covered approximately 70% of the NTSC standard, as shown in Fig. 5b. In fact, this value is not higher than the previously reported PQD CCL^10–12^, which might be related to blue light leakage. In addition, when the color gamut is recalculated by applying the color filter, considering that 128% NTSC is obtained (Fig. S6), it may be concluded that the blue light leakage was not completely prevented by employing blue-light-cutting film.

Figure 5c–e present the monochromatic light emission images of RGB subpixels of the PQD micro-LED display, and Fig. 5f shows the full-color light emission images of the PQD micro-LED display. Our micro-LED display has a 1 mm pixel pitch, that is, a resolution of about 25.4 pixels per inch (PPI), which is comparable to that of outdoor digital signage displays such as video walls. Because the black matrix mold of the micro-LED display is flexible and thin, the proposed color conversion design provides mechanical flexibility, as shown in Fig. 5g. Specifically, the full-color micro-LED display can endure a bending radius of 5 mm without damage.

## Conclusions

In summary, the introduction of silane ligands in PQDs via surface activation by addition of anions enables the preparation of PQD/siloxane composites without aggregation and degradation of PQDs. These composite materials have superior atmospheric stability compared with PQD CCLs prepared *via* conventional ink-jet or vacuum drying processes and can be manufactured to a sufficient thickness to achieve high light conversion efficiency and photopatterning properties. The quantum yields of the green and red PQD/siloxane composites in film form reached 89% and 55%, respectively, and the FWHMs were around 22.7 and 39.8 nm. Using these materials and processes, a full-color micro-LED display with a resolution of about 25.4 PPI on a 1 mm pixel pitch was successfully fabricated.

## Supplementary Information

Supplementary Information.

## Data Availability

The datasets used and/or analyzed during the current study are available from the corresponding author on reasonable request.

## Abbreviations

CCL(s): Color conversion layers
PQD(s): Perovskite quantum dots
PPI: Pixels per inch
QD(s): Quantum dots
PLQY: Photoluminescence quantum yield
PDMS: Polydimethylsiloxane
OA: Oleic acid
OAm: Oleylamine
ODE: 1-Octadecene
MeOAc: Methyl acetate
MPTMS: 3-(Trimethoxysilyl)propyl methacrylate
DPSD: Diphenylsilanediol
TEM: Transmission electron microscopy
PL: Photoluminescence
PI: Polyimide
PCB: Printed circuit board
FTIR: Fourier transform infrared
UV: Ultraviolet
FWHM: Full width at half maximum
