# Supplementary material for: Full-color micro-LED display with photo-patternable and highly ambient-stable perovskite quantum dot/siloxane composite as color conversion layers
Source: Sci Rep. 2023 Mar 24;13:4836. doi: 10.1038/s41598-023-31945-6 (PMC10039071; doi:10.1038/s41598-023-31945-6)
Supplement: Supplementary file 1 — Supplementary Information. [file 41598_2023_31945_MOESM1_ESM.pdf]

Supporting Information

Full-Color Micro-LED Display with Photo-Patternable and Highly Ambient-Stable Perovskite Quantum Dot/Siloxane Composite as Color Conversion Layers

Hyung Cheoul Shim[1],[2],[§],[*], Juho Kim[1],[§], So Yeon Park[1],[§], Bong Sung Kim[1], Bongkyun Jang[1],[2], Hak-Joo Lee[1],[3], Areum Kim[1], Seungmin Hyun[1],[2] and Jae-Hyun Kim[1],[2],[*]

[1] Department of Applied Nano-Mechanics, Korea Institute of Machinery & Materials (KIMM), 156, Gajeongbuk-ro, Yuseong-gu, Daejeon, 34103, Republic of Korea

[2] Department of Nanomechatronics, University of Science and Technology (UST), Daejeon 34113, Republic of Korea

[3] Center for Advanced Meta-Materials (CAMM), 156 Gajeongbuk-Ro, Yuseong-gu, Daejeon 34103, Republic of Korea

[*] indicates the corresponding author

[§] H.-C. Shim, J. Kim and S.-Y. Park contributed equally to this work

[*] Corresponding author: Hyung Cheoul Shim, Jae-Hyun Kim

[*] Tel.: +82-42-868-7143 (H.C. Shim), +82-42-868-7550 (J.-H. Kim),

E-mail address: scafos@kimm.re.kr (H.C. Shim), jaehkim@kimm.re.kr (J.-H. Kim)

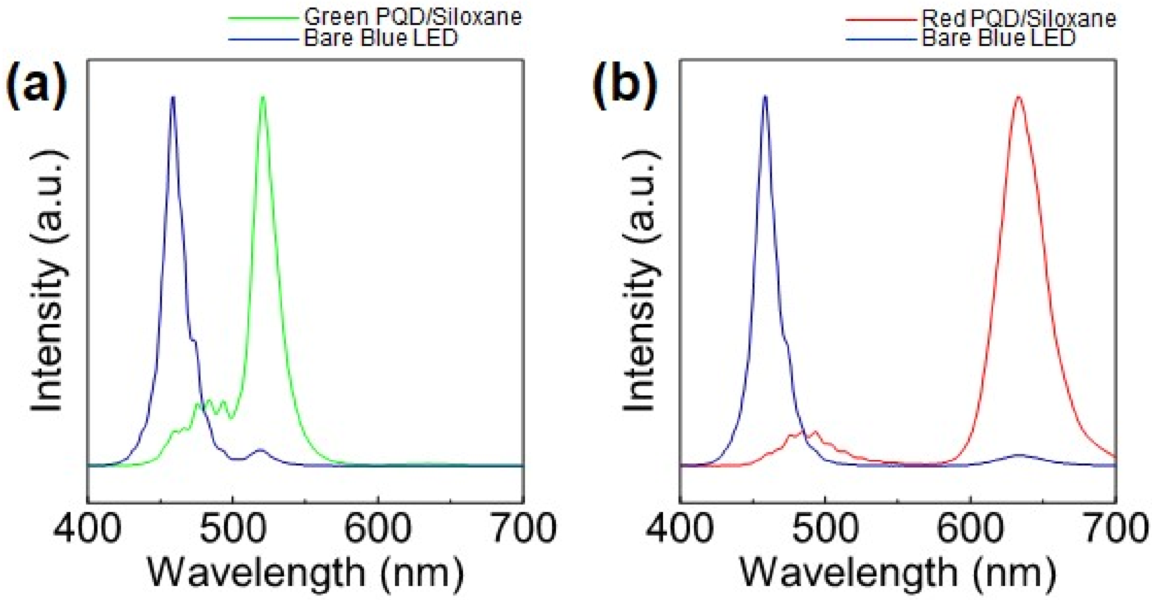

FIG. S1. The PL spectra of (a) green PQD/siloxane and (b) red PQD/siloxane composite with bare blue LED emission without blue light cutting filter.

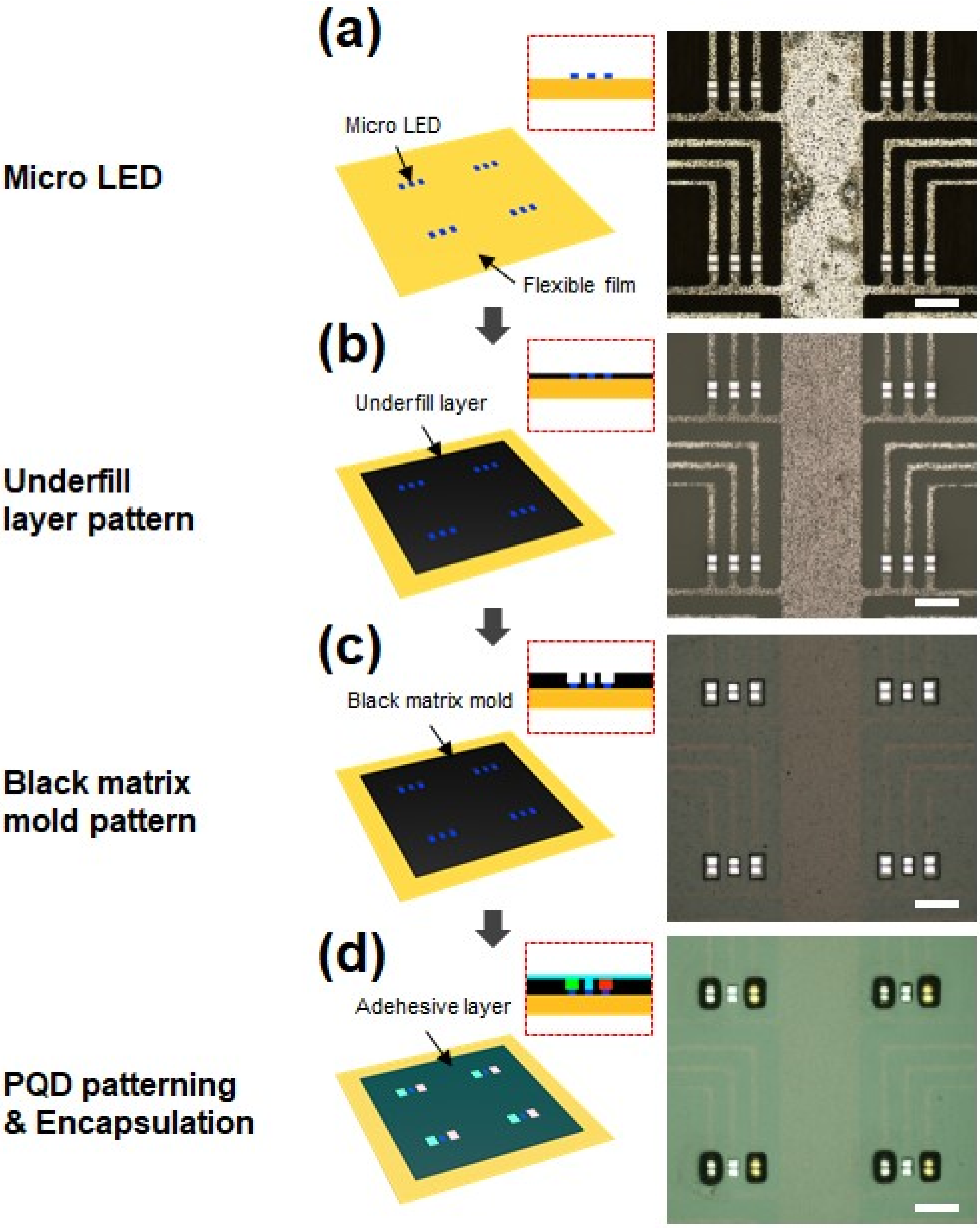

FIG. S2. Schematic illustration and optical microscope image of fabrication procedure for patterned black matrix mold in micro-LED with PQD CCLs.

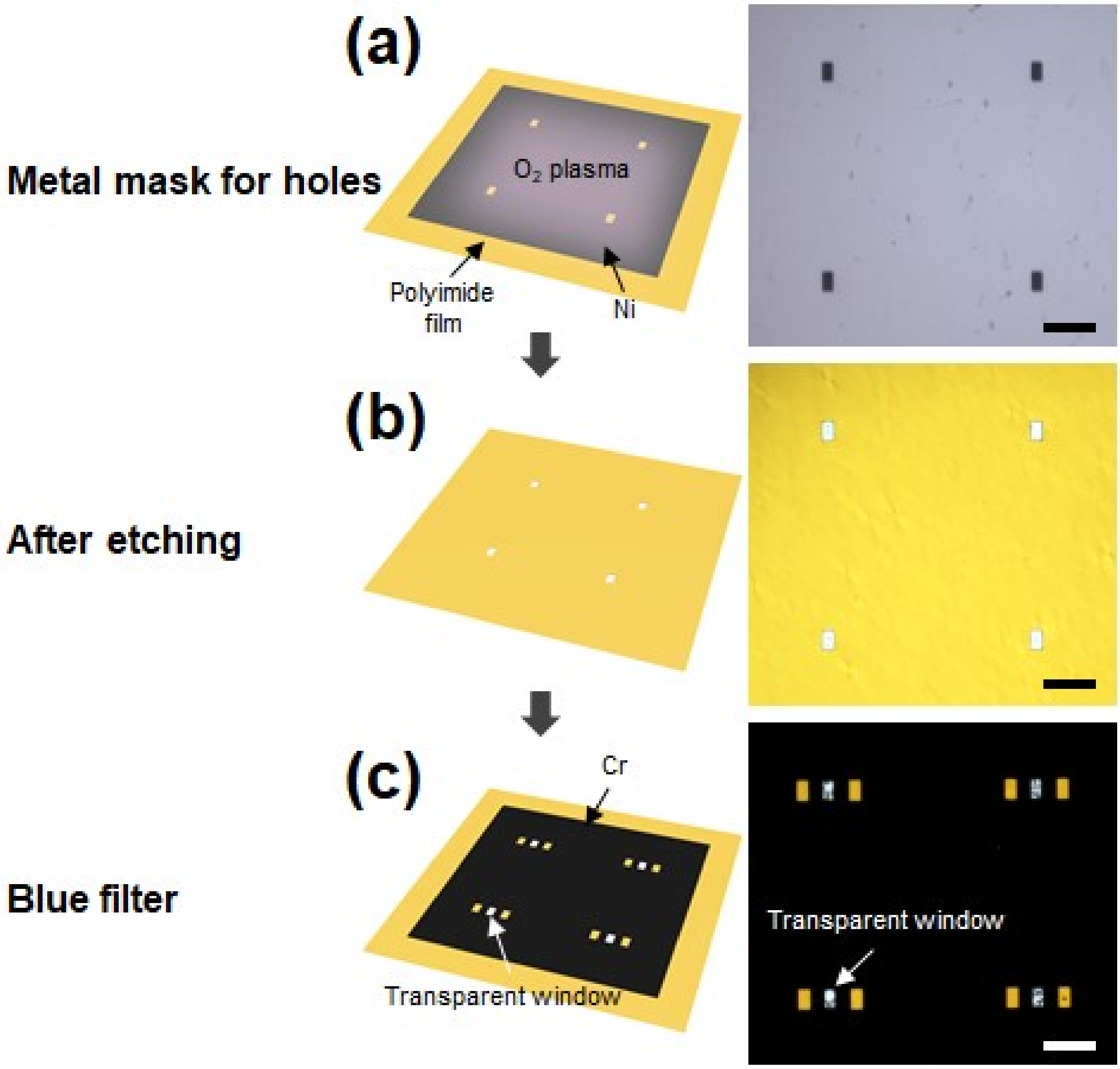

FIG. S3. Schematic illustration and optical microscope image of fabrication procedure for blue light cutting filter.

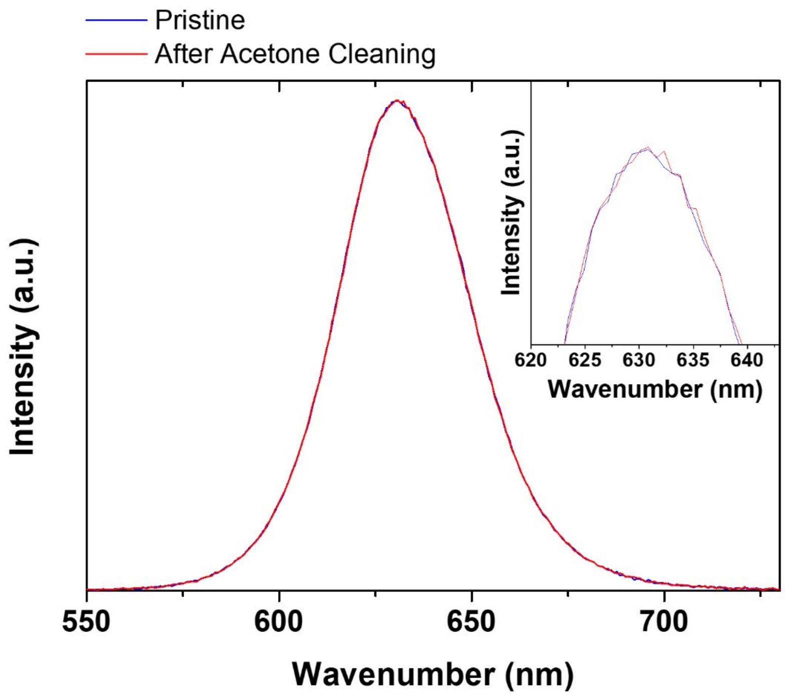

FIG. S4. PL spectra of red perovskite quantum dots (PQDs)/silane composite film before (blue line) and after (red line) acetone cleaning process.

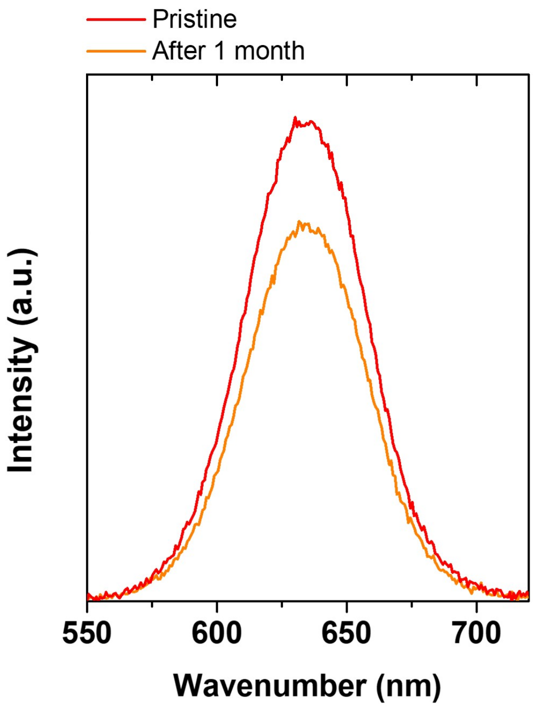

FIG. S5. PL spectra of silane-capped red PQD/siloxane composite. The orange line indicates the PL spectra after one month of storage under ambient condition.

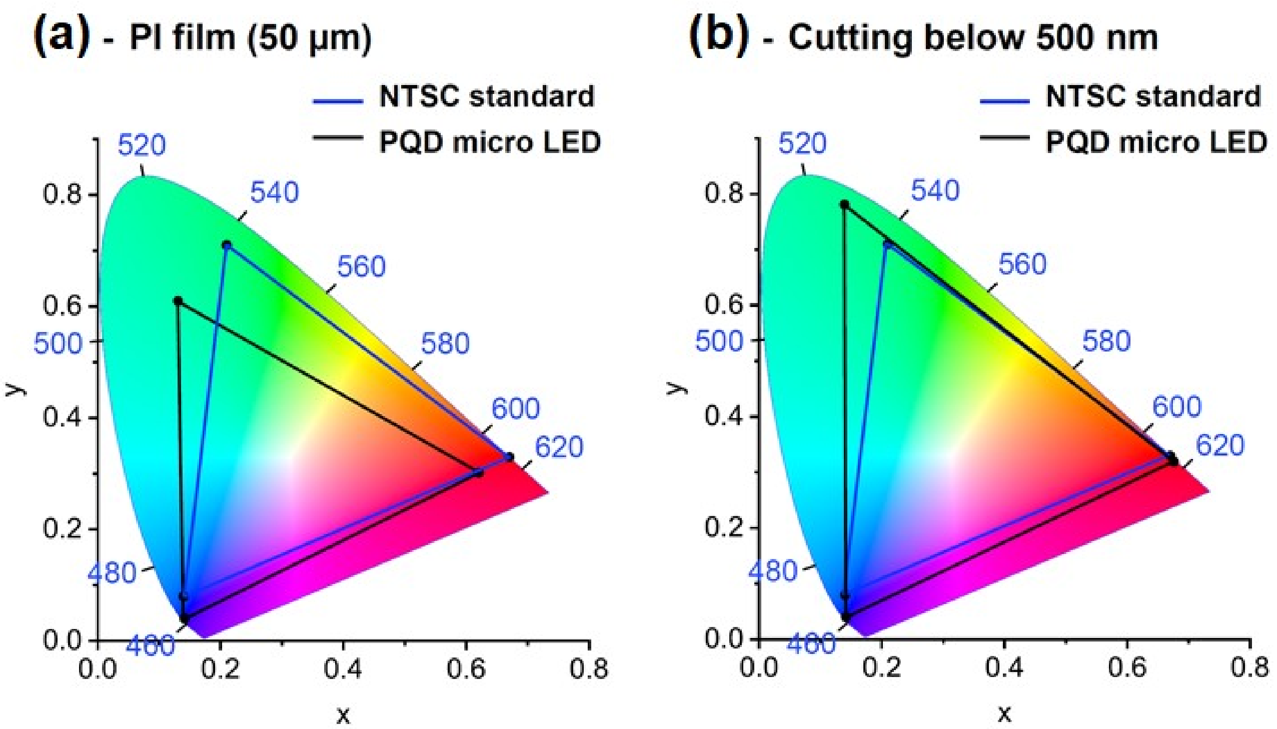

FIG. S6. CIE color coordinates corresponding to the RGB light of (a) as-prepared display and (b) color filter applied display. The black and blue lines show the CIE color coordinates of the PQD micro-LED display and NTSC, respectively.

Materials	PLQY (%)
Oleate-PQD	88.5
Silane-PQD	84.1

Table S1. PLQYs of green PQD solution during ligand exchange process.

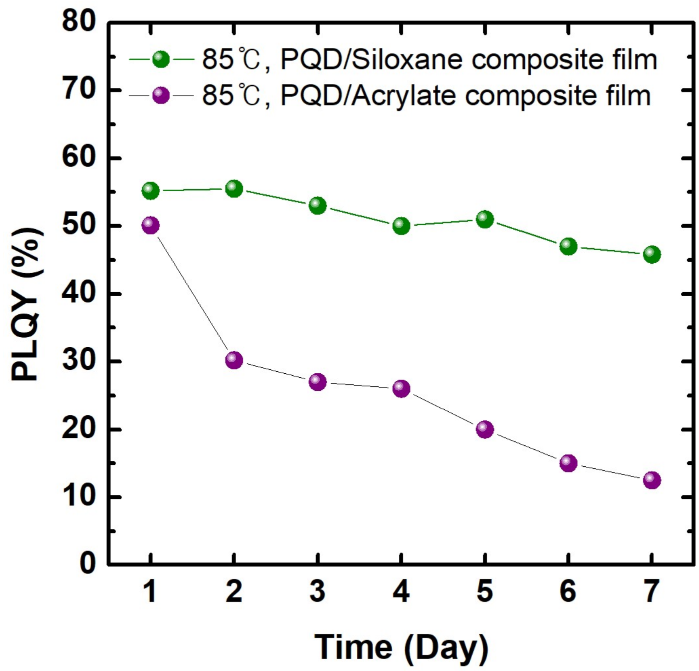

FIG. S7. PLQYs traces of PQD/siloxane and PQD/acrylate composite film upon thermal exposure at 85 °C for a week

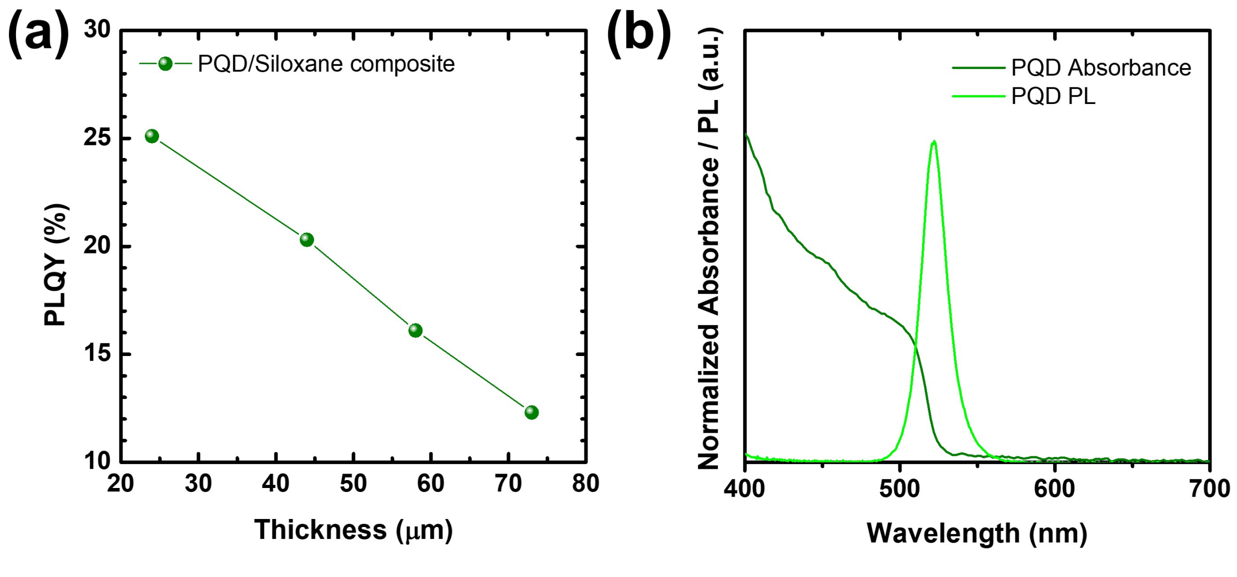

FIG. S8. (a) The PLQYs of the PQD/siloxane composite film with different thickness. (b) Absorption and PL spectra of PQD solution.
